# Effects of the COVID‐19 pandemic on caregiver mental health and the child caregiving environment in a low‐resource, rural context

**DOI:** 10.1111/cdev.13651

**Published:** 2021-09-07

**Authors:** Helen O. Pitchik, Fahmida Tofail, Fahmida Akter, Jesmin Sultana, AKM Shoab, Tarique M. N. Huda, Jenna E. Forsyth, Natasha Kaushal, Tania Jahir, Farzana Yeasmin, Rizwana Khan, Jyoti B. Das, Md. Khobair Hossain, Md. Rezaul Hasan, Mahbubur Rahman, Peter J. Winch, Stephen P. Luby, Lia C. H. Fernald

**Affiliations:** ^1^ Division of Epidemiology, School of Public Health University of California Berkeley California USA; ^2^ Nutrition and Clinical Services Division International Centre for Diarrhoeal Disease Research Bangladesh (icddr,b) Dhaka Bangladesh; ^3^ Infectious Diseases Division International Centre for Diarrhoeal Disease Research, Bangladesh (icddr,b) Dhaka Bangladesh; ^4^ Woods Institute for the Environment Stanford University Stanford California USA; ^5^ Department of International Health Bloomberg School of Public Health, Johns Hopkins University Baltimore Maryland USA; ^6^ Division of Infectious Diseases and Geographic Medicine Stanford University Stanford California USA; ^7^ Division of Community Health Sciences School of Public Health, University of California Berkeley California USA

## Abstract

Early child development has been influenced directly and indirectly by the COVID‐19 pandemic, and these effects are exacerbated in contexts of poverty. This study estimates effects of the pandemic and subsequent population lockdowns on mental health, caregiving practices, and freedom of movement among female caregivers of children 6–27 months (50% female), in rural Bangladesh. A cohort (*N* = 517) was assessed before and during the pandemic (May–June, 2019 and July–September, 2020). Caregivers who experienced more food insecurity and financial loss during the pandemic reported larger increases in depressive symptoms (0.26 *SD*, 95% CI 0.08–0.44; 0.21 *SD*, 0.04–0.40) compared to less affected caregivers. Stimulating caregiving and freedom of movement results were inconsistent. Increases in depressive symptoms during the pandemic may have consequences for child development.

**Abbreviations**
CES‐D: Center for Epidemiologic Studies Depression scale
DID: Difference‐in‐differences
FCI: Family Care Indicators
GEE: generalized estimating equation
LMIC: low‐ and middle‐income country

The COVID‐19 pandemic threatens child development throughout the world as nutrition, routine healthcare, and the ability to provide nurturing care for young children are disrupted (Yoshikawa et al., 2020). In many low‐ and middle‐income countries (LMICs), COVID‐19 lockdowns have resulted in difficulties accessing essential healthcare, reductions in child vaccination rates, and economic effects that are disproportionally felt by poor families (Cash & Patel, 2020).

Studies on the effects of the COVID‐19 pandemic on young children in LMICs to date have been limited to primarily outcomes of mortality and morbidity. The disruptions in routine healthcare and access to food in LMICs induced by the COVID‐19 pandemic have been estimated to result in at least 9.8% additional child deaths per month (Roberton et al., 2020). In addition to mortality, reduced income and food insecurity induced by the COVID‐19 pandemic and resulting government‐mandated mitigation measures including national and regional lockdowns may lead to increases in nutrition‐related morbidities including poor dietary intake, higher disease incidence with longer durations, lasting effects on child growth and development, and higher risk of compromised maternal health and intergenerational transfer of poor nutrition (Akseer et al., 2020; Laborde et al., 2020).

The *nurturing care framework* summarizes the inputs required to promote healthy child development in the first few years of life and includes health, nutrition, security and safety, responsive caregiving, and opportunities for early learning (Britto et al., 2017; World Health Organization et al., 2018). Given that young children rely on their parents or other adults for care, they experience the pandemic and subsequent lockdowns through their caregivers’ ability to provide nurturing and responsive care (Yoshikawa et al., 2020). Social, economic, political, climactic, and cultural contexts affect the enabling environment for caregivers to provide nurturing care (Black et al., 2017). There are multiple components of nurturing care and the enabling environment for caregiver provision of nurturing care that may be affected by the COVID‐19 pandemic and subsequent lockdowns, including caregiving physical and mental health, child health and nutrition, and responsive caregiving (Figure 1; Black et al., 2017).

**FIGURE 1:**
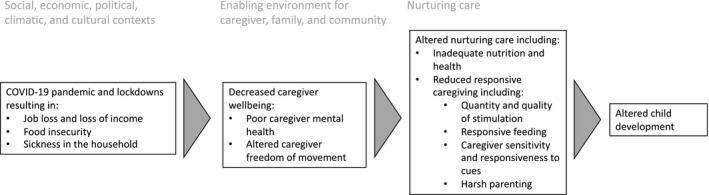
A conceptual diagram for the impact of the COVID‐19 pandemic on child development through the lens of the nurturing care framework

Maternal mental health and the ability to provide stimulating and responsive care are adversely affected when families are facing economic hardship (Black et al., 2017; Herba et al., 2016; Lund et al., 2011). Parenting quality is a mediator between maternal mental health and child outcomes (Black et al., 2007; Stein et al., 2014). Poor maternal mental health can affect children through multiple pathways including altered maternal‐child interactions, decreased early childhood attachment, and harsh punishment (Herba et al., 2016). Mothers who are depressed are less responsive to their infant children, and less likely to be engaged in responsive stimulation (Black et al., 2007; Esposito et al., 2017). Further, the relationship between maternal mental health and stimulating parenting behaviors is cyclic, and responds dynamically to child temperament and behavior (Bagner et al., 2013). Mothers who engage in responsive stimulation may be at a decreased risk of developing depressive symptoms, possibly due to improved mental health following rewarding experiences with the child (Singla et al., 2015).

Women's empowerment affects both maternal and child health and is comprised of multiple dimensions including resources, agency, and achievements (Glennerster et al., 2018; Pratley, 2016). Freedom of movement is one indicator of women's empowerment that may have been impacted as a result of COVID‐19 lockdowns and their sequelae (Mahmud et al., 2012; Schuler & Hashemi, 1994). Freedom of movement means women are willing and able to travel to health centers, friends and relatives’ houses, outside of the village, and to the market on their own. However, during the COVID pandemic, more frequent trips outside of the home may also represent risky behaviors.

There have been notable gains in maternal and child health in Bangladesh in the 21st century, including large decreases in under five, infant, and maternal mortality, as well as increases in life expectancy at birth (Chowdhury et al., 2013). These gains have been partially attributed to the structure and reach of the country's health system (Chowdhury et al., 2013). However, Bangladesh still faces a high prevalence of poverty, income inequality, undernutrition, and stunting. Bangladesh is a country with a population of over 160 million, 23% of whom live in poverty (Bangladesh Bureau of Statistics, 2016; World Bank, 2018). A substantial number of children in Bangladesh are stunted (31%), underweight (22%), or wasted (8%), and 40% of women between age 15–40 experience iron‐deficiency anemia (Bangladesh Bureau of Statistics & UNICEF Bangladesh, 2019; National Institute of Population Research and Training et al., 2020). Despite exposure to multiple risk factors for poor child development, 72% of 3‐ and 4‐year old rural children are developmentally on track in the social‐emotional domain as assessed by the early child development index (Bangladesh Bureau of Statistics & UNICEF Bangladesh, 2019). In terms of literacy and numeracy, however, only 29% of 3‐ and 4‐year old children are developmentally on track (Bangladesh Bureau of Statistics & UNICEF Bangladesh, 2019).

The United Nations Convention for Rights of the Child is a human rights treaty that protects and promotes the civil, political, economic, social, health, and cultural rights of children (UN General Assembly, 1989). Bangladesh signed onto the convention in 1990, and is joined by the vast majority of the world's nations as signatories. The convention states “States Parties shall ensure to the maximum extent possible the survival and development of the child” (UN General Assembly, 1989). Further, the Sustainable Development Goals explicitly include quality child development as a focus in goal 4.2, which is “By 2030, ensure that all girls and boys have access to quality early childhood development, care and pre‐primary education so that they are ready for primary education” (United Nations, 2015). The current work is guided by these principles, and we focus on identifying children who may be at increased risk for altered developmental trajectories in the context of the COVID‐19 pandemic. Though not all children experiencing risk factors have compromised development, the duration, co‐occurrence, and magnitude of these risks contribute to increased probability of delay (Wachs, 2012).

In response to the pandemic, the Government of Bangladesh shut down schools, business, and other institutions between March and May 2020. Some businesses and other institutions re‐opened, but schools and educational institutions continued to be closed until May 23, 2021 with a re‐opening planned for thereafter. Recent research demonstrated that in May and June 2020 the median income of families in a rural area just outside the capital city of Dhaka fell to just over 25% of what it was 1 year previously, severe food insecurity increased from 6% to 36%, and maternal depressive symptoms increased (Hamadani et al., 2020). Intimate partner violence is a risk factor for maternal depression, and in May and June, over half of women who reported experiencing intimate partner violence reported an increase since the shut‐down for COVID‐19 began (Gelaye et al., 2016; Hamadani et al., 2020).

## The present study

The primary aim of this research was to assess the effects of the COVID‐19 pandemic and related lockdowns on risk factors for poor early child development including maternal mental health and caregiver stimulation in the home in a large sample of families in rural Bangladesh. Understanding the effects of the COVID‐19 pandemic and related lockdowns on risk factors for caregivers will contribute to the understanding of the impact of COVID‐19 on child development. A secondary aim was to explore the relationship between the pandemic and caregiver freedom of movement.

## METHOD

### Sample recruitment and selection

All data come from assessments done as part of the Research on Integration of Nutrition Early Childhood Development and WASH intervention delivered through the Government health system in Bangladesh (RINEW‐G) study, which was set in Chatmohar. Chatmohar sub‐district is located in Pabna district of Central Bangladesh, consists of 11 rural unions and one urban municipality, and had a population of 291,121 recorded at the most recent population census in 2011 (Bangladesh Bureau of Statistics, 2011). The primary occupation of employed residents is in agriculture, other less common occupations are small business owners, or salaried government, private business, or non‐governmental organization workers. In the most recent census, less than half of the population over 7 years old in Chatmohar was literate (46%), and 83% of the population lived in houses with no permanent structure (‘katcha’; Bangladesh Bureau of Statistics, 2011). The majority of the population of Chatmohar is Muslim (96%), and 96% of the households own the dwelling they live in (Bangladesh Bureau of Statistics, 2013).

**FIGURE 2:**
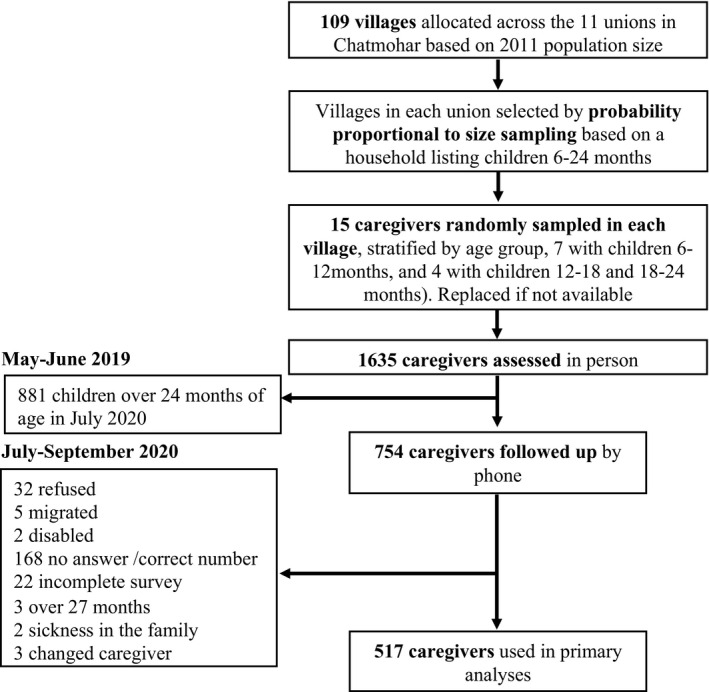
Study sample

The study team collected data at two time points: a baseline (“pre‐COVID”; May 18–June 22, 2019), and a follow‐up, 4 months after the first case of COVID was detected in Bangladesh (“mid‐COVID”; July 11–September 16, 2020). The in‐person pre‐COVID assessments consisted of a sample of 1635 primary caregivers of children 6–24 months old living in 109 rural villages of Chatmohar, selected through multi‐stage sampling (Figure 2; Supporting Information: page 1). Exclusion criteria were caregivers who were not planning on living in Chatmohar for the following year and caregivers or children with physical or cognitive disabilities. At the mid‐COVID assessment, the study team contacted 754 caregivers from the original sample who had children under 24 months of age in July 2020 (Figure 2). An additional cross‐sectional sample was also recruited in order to gather information during the mid‐COVID timepoint across the original age range of 6–24 months. The majority of this cross‐sectional sample were caregivers of children between 6 and 18 months of age, and lived in the same villages sampled during the pre‐COVID assessment (Figure S1a,b). This mid‐COVID cross‐sectional sample was used in the present study in a supplementary analysis examining correlations between exposures and outcomes across the larger sample and age range.

Before data collection, all participants provided written consent during the pre‐COVID in‐person visit, and verbal consent during the mid‐COVID phone survey.

### Measures

Pre‐COVID assessments were conducted in person in participant's homes. Mid‐COVID assessments were conducted over the phone. Measures collected at both time points are described below.

Training for data collection lasted 15 days for the pre‐COVID assessment, and 8 days for the mid‐COVID assessment. Training included instruction, role‐play practice with peers, and practice with sample participants. During both assessment time points, supervisors conducted ongoing quality control for 5% of the sample, with supervisors observing assessments (either watching in person or listening on the phone) to provide feedback following the assessment if needed.

#### COVID‐19 experiences

The questionnaire administered during the mid‐COVID assessment included modules to assess experiences during the COVID pandemic (Table S1). Questions included changes in the number of household members, interactions with the target child, changes made in the household due to COVID‐19, indicators of food insecurity, changes in household economic status and coping mechanisms, and sickness in the household. These questions were developed through an iterative process including qualitative interviews and pre‐testing, and used some questions from a survey previously done in Bangladesh (survey development described in Supporting Information: page 2; Lopez‐Pena et al., 2020).

Using the survey responses, we characterized the effects of COVID‐19 across three domains: food security, economic status, and health. Participants were split into categories of more or less affected in each of these three domains. For the food security domain, participants were categorized as more affected if they answered that they were negatively affected in response to two or more of the following questions: (1) Was your household able to buy essential food items over the past 7 days; (2) in the last 7 days, are you consuming less food when compared to the same time last year, and is that reduction due to COVID‐19 pandemic; (3) in order to cover household's basic needs, participants have had to reduce the number or size of meals for some household members, or (4) rely on less preferred and less expensive foods. For the economic impact domain, participants were characterized as more affected if both (1) since April 2020 (the start of the COVID‐19 lockdown in Bangladesh), the main earning member of the household lost their job/income source and (2) the status of the household's current income was reported as none or reduced compared to April 2020. For the health domain, households were categorized into ones where either the respondent or any household member had been sick with symptoms of fever, cough, cold, loss of taste/smell, shortness of breath or difficulty breathing, or COVID‐19 between April 2020 and the time of the mid‐COVID assessment.

#### Outcomes

Outcomes were measured at both the pre‐COVID and mid‐COVID time points. Maternal depressive symptoms were assessed using the Center for Epidemiologic Studies Depression scale (CES‐D), which consists of 20 questions asking about the number of days each participant experienced depressive symptoms in the past week. Each question was scored on a Likert scale, including 0 (0 days), 1 (1–2 days), 2 (3–4 days), and 3 (5–7 days), and individual question scores were summed to a total range of 0–60, with higher scores representing more depressive symptoms (Radloff, 1977). The scale reliability of the CES‐D score in the sample was relatively high (*α* = .83 at the pre‐COVID assessment and .84 at the mid‐COVID assessment for the cohort sample).

Child stimulation in the home was assessed with the Family Care Indicators (FCI), which has been used previously in Bangladesh (Hamadani et al., 2010), and consist of two subscales, stimulating caregiving practices and the variety of play materials available in the home. The stimulating caregiving practices subscale asks if the child's mother, father, or any adult (>15 years old) has engaged with the child in six stimulating activities in the previous 3 days. We conducted the primary analysis of this outcome with maternal reported participation in stimulating caregiving practices (range 0–6) as the mother was the respondent and we hypothesized that this would be the most accurate report. The variety of play materials subscale consists of questions about the variety of play materials that the child has played with in the past 30 days (range 0–6). The correlation between the play activities and play materials subscales of the FCI was 0.30 during the baseline assessment and 0.32 at the mid‐COVID assessment.

Freedom of movement for the primary caregiver was measured with questions about experiences going to the market, a medical facility, outside of the village, and to a paternal home or friends or relatives’ home in the last 6 months. Each participant was given a freedom of movement score, which included one point if the respondent had attended each place in the last 6 months, and an additional point if the respondent had been to this place alone in the past 6 months, with a total range of 0–8. These questions were adapted from a scale used previously in Bangladesh (Biswas & Kabir, 2004). The scale reliability of freedom of movement scores was 0.61 and 0.64 during the pre‐ and mid‐COVID assessments, respectively.

#### Covariates

Information on the family's socioeconomic status was collected at both time points with questions about monthly income (separated into tertiles), household assets, household size (categorized into 2–3, 4, 5, 6, 7+ household members), maternal education (categorized into none, less than primary school, completed school, and completed secondary school), and housing materials (indicators were made for if the household had a concrete floor or brick walls). Caregivers were asked if they get to spend their own money independently, and the responses were categorized into a binary response where responses of “no” and “no independent money” were combined. Healthcare access and health‐seeking behavior were measured through the number of antenatal care visits that each participant reported attending prior to their most recent pregnancy, and was categorized into above or below 4, the recommended number of visits.

At the pre‐COVID assessment, we also measured some indicators of nutrition and WASH status, which were used in the comparison of pre‐COVID characteristics for the difference‐in‐differences samples. Maternal and child dietary diversity was assessed by asking about all foods consumed in the previous 24 h. Caregivers who consumed at least 5 of 10 food groups were considered having met minimum dietary diversity. Children's dietary diversity was assessed with 8 food groups, including breastmilk, and were considered having met the minimum dietary diversity threshold if they had consumed 5 or more of the 8 groups (FAO & FHI 360, 2016; World Health Organization, 2017). The status of WASH facilities in the household was assessed through observation, and the presence of a handwashing station with water and soap or a soapy water bottle within 20 m of the cooking facility, and of a clean, functional, hygienic latrine in the household was assessed.

### Statistical analysis

Descriptive statistics included summaries of the distribution and histograms for continuous variables, and tabulations of proportions for categorical variables. Our primary analysis to examine the effects of the COVID‐19 pandemic and subsequent lockdowns on risk factors for poor child development was a difference‐in‐differences analysis with the longitudinal cohort assessed at both the pre‐COVID and mid‐COVID time points. Our supplementary analysis consisted of a cross‐sectional comparison for all participants in the mid‐COVID sample. For both analytic strategies, we examined four outcomes of interest: our primary outcomes of maternal depressive symptoms, stimulating caregiving practices, and varieties of play materials, and a secondary outcome of caregiver freedom of movement.

For the difference‐in‐differences analysis, we compared changes in outcomes over time for those the cohort sample who were more versus less affected on the food security, economic, and health domains at the mid‐COVID assessment. We use generalized estimating equations (GEEs) controlling for an indicator of village membership, and accounting for repeated observations within the cohort sample.

For the supplementary analysis on the full cross‐sectional mid‐COVID assessment, we conducted an adjusted cross‐sectional comparison of outcomes at the mid‐COVID assessment, comparing families that reported being more versus less affected on the food security, economic, and health domains at the mid‐COVID assessment. We estimate differences in outcomes between groups using a generalized linear model with standard errors adjusted for clustering by village.

In both analyses, we controlled for theoretical confounders of child age, maternal education, income, antenatal care, control over assets, household size, child sex, and housing materials. For the difference‐in‐differences analyses, we controlled for baseline values of these variables, and for the cross‐sectional analyses, we controlled for concurrent values, as the sample included some cross‐sectional participants who do not have baseline measurements. In all analyses, we additionally controlled for attendance at intervention sessions. Further, in the difference‐in‐differences analyses with maternal depressive symptoms or home stimulation outcomes, we control for baseline caregiver freedom of movement.

For the maternal depressive symptoms outcome, we expected the distribution of scores to be non‐normal, with a right skew. We conducted sensitivity analyses with median regression in addition to our primary analysis. As GEE models allowed us to account for repeated measures, we prefer the GEE results and present these as our main findings.

In order to estimate the effects on child development, we stratified the population into more and less depressed using a cut‐off value of 16 for the CES‐D score. In the United States, a CES‐D score cut‐off of 16 has been validated to indicate higher risk of clinical depression (Lewinsohn et al., 1997). As this cut‐off has not been validated in Bangladesh, we adopted the continuous CES‐D score in our primary analyses. We took the cut‐off value as a proxy for more depressive symptoms to compare our results to the literature looking at the relationship between caregiver depression and child development. We conducted a difference‐in‐differences analysis with a binary outcome indicating CES‐D score 16 and above or 15 and under. We estimate this model with a GEE with an identity link to estimate risk differences, accounting for repeated observations by village. Using our results from this analyses and meta‐analyzed estimates of the relationship between maternal depressive symptoms and child development outcomes, we estimated the burden of increased risk for poor child development in rural Bangladesh. Statistical analyses were performed in Stata 14 and R (Version 4.0.2).

Given the longitudinal study design, and multiple analyses undertaken for robustness, we viewed the analyses on maternal depressive symptoms, stimulating activities with the child, and variety of stimulating play materials work as confirmatory. Due to the use of only a single measure to draw inference on a larger construct, we considered the analyses that include the outcome of caregiver freedom of movement to be exploratory.

## RESULTS

### Participants

The study team collected data on 1635 caregivers of children 6–24 months of age during the pre‐COVID assessment, and 754 of these children were under 24 months in July 2020, and followed‐up (Figure 2). A total of 523 households from the pre‐COVID sample were reached for complete mid‐COVID data collection (69% of those attempted). Six participants were excluded from the analytic sample, three because their child was over 27 months of age at the time of assessment, and three for having a different caregiver assessed at the second time point. In all, 517 caregivers comprise the cohort sample (Figure 2). The cohort sample that was followed up was not different from those who were attempted to be followed up and were not reached across multiple demographic factors (Table S2). An additional cross‐sectional sample of 1176 participants was collected during the mid‐COVID assessment, 54% of those contacted, and are used in a supplemental analysis (Figure S1a).

Most caregivers in the cohort sample had completed primary education, the mean child age was 8.55 months (*SD* 1.8), and approximately one‐fifth of the sample had a cement floor, brick walls, and a refrigerator (Table 1).

**TABLE 1:** Demographic characteristics of the cohort during the pre‐COVID assessment

	% (*n*) or mean ± *SD*
	Cohort (*n* = 517)
Caregiver characteristics	
Completed primary education (6+ years)	69% (369)
Currently pregnant	1% (6)
Muslim	98% (510)
Child characteristics	
Age (in months)	8.55 ± 1.8
<6 months	7% (34)
6–12 months	93% (482)
12–18 months	<1% (4)
Female	51% (263)
Household characteristics	
Household size	5.23 ± 1.80
Number of children <15 years under care	1.9 ± 0.80
Has cement floor	19% (98)
Has brick walls	23% (123)
Has electricity	99% (514)
Has refrigerator	22% (112)

### COVID‐19‐related responses during the mid‐COVID assessment

When asked what changes they had made due to the COVID‐19 pandemic (no prompt given), most respondents reported that they were washing their hands more (66%, *n* = 334), and cleaning the household and outdoor space more (61%, *n* = 316; Table 2). Some said that they were interacting less with people outside the household (19%, *n* = 98), had restricted their movement (16%, *n* = 83), or were wearing a mask when going outside the house (15%, *n* = 80; Table 2). About a fifth (16%, *n* = 81) of participants said that they had made no changes (Table 2). Most participants reported that they interacted with their child (talking, playing, or spending time) the same amount compared to before the COVID‐19 lockdown (53%, *n* = 274), while 41% (*n* = 210) said that they interacted more, and 6% (*n* = 33) said less (Table 2). Of those who said they interacted more, the most common reason (77%, *n* = 162) was spending more time in the household due to restricted movement outside the house (data not shown). Of those that said they interacted less, the majority (48%, *n* = 16) said this was because they had more household chores (data not shown). Just under a quarter of participants (22%, *n* = 114) had the number of household members change since April, with most of these participants having their household size increased with an adult or child moving in (87%, *n* = 99; Table 2). Most of these moves were temporary with 17% of the families who reported having additional members still staying at the time of the survey (data not shown). Patterns of COVID‐related responses in the full mid‐COVID sample were similar to those in the cohort (data not shown).

**TABLE 2:** COVID‐19‐related responses in the cohort sample at the mid‐COVID assessment

Since the shutdown due to COVID‐19:	Cohort (*n* = 517)	
	%	*n*
Household composition		
Any changes to number of people in HH	22	114
Increased HH members (*n* = 114)	97	99
Interaction with child compared to prior to April 2020		
Same interaction	53	274
More interaction	41	210
Less interaction	6	33
Changes made due to COVID‐19 (free response)		
More handwashing	66	334
More cleaning of household and outdoor space	61	316
Interacting less with people outside the household	19	98
Restricted movement	16	83
Wearing a mask while outside	15	80
No changes made	16	81
Food security		
Not able to buy essential food last 7 days	23	122
Reason (select multiple): HH has less money (*n* = 122)	95	116
Reason (select multiple): items cost more (*n* = 122)	14	17
In the last 7 days, consuming less food when compared to the same time last year, due to COVID‐19	44	230
In the past 7 days, did you or your household members use any of the following to cover your household's basic needs? (select all)		
Reduce the number or size of meals for some HH members^a^	29	152
Rely on less preferred and less expensive foods^a^	39	202
Use cash or bank savings	40	208
Take loan from someone else	34	176
Borrow food or ask for help from a friend, relative, or neighbor	17	87
Look for ways to earn additional money	15	80
Sell assets	8	39
Rely on government or NGO assistance	3	16
No strategy used	20	104
Two or more indicators of food insecurity^b^	41	214
Economic impacts		
Change of employment for any household member since April	68	351
No job or income source of the main earning member of HH	42	219
Status of HH income since April 2020		
No HH income since April	10	55
Reduced HH income since April	71	366
Same HH income since April	15	79
Increased HH income since April	3	17
Both main earning member of HH lost job, and reduced HH income	40	208
Health		
Any HH member sick with COVID‐19 symptoms^a^	35	181
Respondent sick with COVID‐19 symptoms^a^	15	77
Family member sick with COVID‐19 symptoms^a^	27	138

Abbreviation: HH, household.

^a^ Fever, cough, cold, loss of taste/smell, shortness of breath, difficulty breathing, COVID‐19.

^b^ Two or more of: In the past 7 days (1) reduced the number or size of meals or (2) relied on less preferred and less expensive foods to cover basic household needs, (3) not able to buy essential food items, (4) consumed less food compared to the same time last year, due to COVID‐19.

Ten percent of families (*n* = 55) reported that the family had earned no income since April, and an additional 71% (*n* = 366) reported a reduced income compared to prior to the start of the pandemic (Table 2). Over a third (42% *n* = 219) of participants reported that since the COVID‐19 shutdown, there was a change of employment where the main earning member of the household lost their job or income source (Table 2). A total of 40% (*n* = 208) of families experience both a loss of job for the main earning member in the household, and a reduction in income (Table 2). In the 7 days prior to the assessment, just less than one‐third of families (29%, *n* = 152) reduced the number or size of their meals to cover basic household needs, over a third (39%, *n* = 202) relied on less preferred and less expensive foods, and almost a quarter (23%, *n* = 23) were not able to buy essential food items (Table 2). Almost half (44%, *n* = 230) of participants reported consuming less food when compared to the same time last year, and attributed the change to the COVID pandemic (Table 2). Overall, 41% (*n* = 214) of participants reported two or more of these indicators of food insecurity, and 67% of those who experienced more food insecurity also experienced a loss of job and a reduced income (Table 2). When participants were asked if they or household members had been sick with symptoms of fever, cough, cold, loss of taste or smell, shortness of breath or difficult breathing, or COVID‐19, 15% (*n* = 77) or participants reported being sick, and 27% (*n* = 138) reported that their family members had been sick (Table 2). Overall 35% (*n* = 181) of participants reported either they or their household members had been sick with these symptoms since April 2020 (Table 2).

When comparing the pre‐COVID and mid‐COVID time points in the cohort sample, on average, after adjusting for covariates, there was no difference in caregiver depressive symptoms between the two time points (−0.55, 95% CI: −1.30 to 0.21; Table 3). Play activities and play materials scores were both increased over time (mean differences for play activities: 1.25 (1.08 to 1.42); play materials 1.66 (1.52 to 1.79)), and the caregiver freedom of movement score decreased over time (−0.41, −0.58 to −0.25; Table 3).

**TABLE 3:** Differences in child‐development risk factors in the cohort comparing pre‐ and mid‐COVID time points (*n* = 517)

Outcome	Mean ± *SD*		Adjusted difference
	Pre‐COVID	Mid‐COVID	
CES‐D score	13.4 ± 8.7	12.8 ± 9.2	−0.55 (−1.30 to 0.21)
FCI play activities	2.5 ± 1.51	3.8 ± 1.6	1.25 (1.08 to 1.42)
FCI play materials	1.2 ± 1.1	2.8 ± 1.2	1.66 (1.52 to 1.79)
Freedom of movement score	3.5 ± 1.6	3.1 ± 1.8	−0.41 (−0.58 to −0.25)

Note

FCI: Family Care Indicators, the play activities subscale is a sum score of the number of play activities that the caregiver participated in with the child in the previous 3 days (0–6); the play materials subscale is the number of different types of play materials the child has played with in the past 30 days (0–6); CES‐D: Center for Epidemiologic Studies 20 question Depression questionnaire (0–60), and higher scores indicate more depressive symptoms experienced; Freedom of movement score is a sum score with one point for attending each of the following four places in the last 6 months, and an additional point if that location was attended alone: the market, medical facility, outside the village, paternal home or the home of a friend or a relative (0–8); Adjusted differences are adjusted for village, child age category, maternal education, income, antenatal care, control over assets, household size, child sex, housing materials.

### Difference‐in‐differences analysis

We compared changes over time in risk factors for poor child development between those in the cohort who reported being more and less affected across the domains of food security, economic status, and health during the mid‐COVID assessment through difference‐in‐differences analyses. The risk factors we looked at were caregiver mental health, stimulating play activities and play materials, and caregiver freedom of movement. There were some differences in observed baseline characteristics between the more and less exposed groups on each of the three domains, with the most differences in pre‐COVID characteristics for the comparison between the caregivers more and less food insecure at the mid‐COVID assessment (Table 4). We adjusted for differences at the caregiver, child, and household levels in the difference‐in‐differences analyses.

**TABLE 4:** Pre‐COVID characteristics of participants stratified by the food security, economic, and health domains at the mid‐COVID assessment

	Food insecure			Lost job and reduced income			Any household member sick		
	Less (*n* = 303)	More (*n* = 214)	*p*‐value^a^	No (*n* = 309)	Yes (*n* = 208)	*p*‐value^a^	No (*n* = 336)	Yes (*n* = 181)	*p*‐value^a^
Caregiver									
Completed primary education^b^	224 (74%)	134 (63%)	.008	212 (69%)	146 (70%)	.775	223 (66%)	135 (75%)	.067
Currently pregnant	3 (1%)	3 (1%)	.992	3 (1%)	3 (1%)	.946	4 (1%)	2 (1%)	1
Children <15 years	1.8 ± 0.8	2.0 ± 0.8	.142	1.9 ± 0.8	1.8 ± 0.8	.260	1.9 ± 0.8	1.8 ± 0.8	.191
CES‐D score	12.5 ± 8.2	14.5 ± 9.3	.012	13.8 ± 9.0	12.7 ± 8.4	.159	12.4 ± 8.3	14.9 ± 9.3	.002
Freedom of movement^c^	3.7 ± 1.7	3.3 ± 1.5	.036	3.6 ± 1.8	3.5 ± 1.5	.492	3.5 ± 1.7	3.6 ± 1.5	.558
Spend own money	191 (63%)	101 (47%)	<.001	178 (58%)	114 (55%)	.59	185 (55%)	107 (59%)	.427
Caregiver MDD^d^	238 (79%)	150 (70%)	.037	238 (77%)	150 (72%)	.246	250 (74%)	138 (76%)	.723
Child									
Age (months)	8.6 ± 1.9	8.5 ± 1.8	.297	8.6 ± 1.8	8.5 ± 1.9	.579	8.6 ± 1.8	8.4 ± 1.8	.169
Female	144 (48%)	112 (52)	.323	150 (49)	106 (51%)	.653	176 (52%)	80 (44)	.092
Child MDD^e^	34 (12%)	19 (10)	.571	33 (11)	20 (10%)	.885	40 (12%)	13 (7%)	.126
FCI Play activities	2.6 ± 1.4	2.5 ± 1.6	.496	2.5 ± 1.5	2.6 ± 1.5	.29	2.5 ± 1.5	2.7 ± 1.5	.153
FCI Play materials	1.3 ± 1.2	1.1 ± 1.1	.044	1.2 ± 1.2	1.1 ± 1.1	.707	1.2 ± 1.2	1.0 ± 1.1	.062
1+ children's books	13 ± 4.3	5 ± 2.3	.342	8 ± 2.6	10 ± 4.8	.269	17 ± 5.1	1 ± 0.6	.016
Household									
Household size	5.3 ± 1.9	5.1 ± 1.6	.151	5.3 ± 1.9	5.1 ± 1.7	.203	5.2 ± 1.7	5.2 ± 2.0	.985
Has refrigerator	83 (27%)	29 (14%)	<.001	79 (26%)	33 (16%)	.012	74 (22%)	38 (21%)	.874
Has television	162 (54%)	96 (45%)	.066	154 (50%)	104 (50%)	1	171 (51%)	87 (48%)	.602
Has brick walls	82 (27%)	39 (18%)	.026	79 (26%)	42 (20%)	.19	75 (22%)	46 (25%)	.494
Has cement floor	69 (23%)	28 (13%)	.008	71 (23%)	26 (13%)	.004	61 (18%)	36 (20%)	.716
Lowest income tertile	131 (43%)	128 (60%)	<.001	149 (48%)	110 (53%)	.059	166 (49)%	93 (51%)	.538
Hygienic latrine	71 (23%)	41 (19%)	.292	61 (20%)	51 (25%)	.236	68 (20%)	44 (24%)	.337
Handwashing station	65 (22%)	25 (18%)	.006	57 (18%)	33 (16%)	.522	63 (19%)	27 (15%)	.33
Attendance									
1 or more sessions attended	165 (55%)	111 (52%)	.623	179 (58%)	97 (47%)	.015	180 (54%)	96 (53%)	.981

Note

All data mean ± *SD* or *n* (%); FCI: Family Care Indicators, the play activities subscale is a sum score of the number of play activities that the caregiver participated in with the child in the previous 3 days (0–6); the play materials subscale is the number of different types of play materials the child has played with in the past 30 days (0–6); CES‐D: Center for Epidemiologic Studies 20 Question Depression questionnaire, scores range from 0 to 60, with higher scores indicating more depressive symptoms experienced.

Abbreviation: MDD, minimum dietary diversity.

^a^ *p*‐values are for comparisons between groups within domains with chi‐square tests for binary or categorical variables or *t*‐tests for continuous variables.

^b^ 6+ years of education.

^c^ Caregiver freedom of movement score (0–8).

^d^ Caregiver reported eating 5 or more food groups in the last 24 h, out of the following 10 groups: grains, legumes, nuts and seeds, dairy products, animal flesh foods, eggs, vitamin A‐rich fruits and vegetables, other vitamin A‐rich fruits and vegetables, other vegetables, other fruits.

^e^ Children >6 months reported eating 5 or more food groups in the last 24 h, out of the following groups: breast milk, grains, legumes, dairy products, animal flesh foods, eggs, vitamin A‐rich fruits and vegetables, other fruits and vegetables.

**FIGURE 3:**
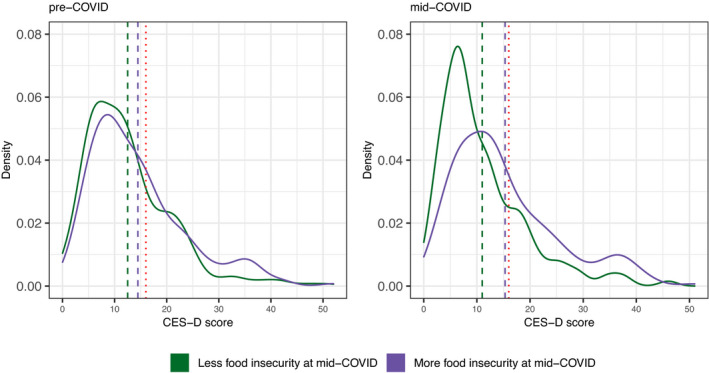
Kernel density plot of CES‐D scores comparing those who have more versus less indicators of food insecurity at pre‐ and mid‐COVID time assessments. Colored dashed lines correspond to mean CES‐D scores in each group; red dashed line indicates CES‐D score of 16. CES‐D, Center for Epidemiologic Studies Depression scale

A kernel density plot of unadjusted CES‐D scores at the pre‐COVID and mid‐COVID time points stratified by experiences of food security during the mid‐COVID assessment displays differences in the distributions for each group over time, illustrating a shift in the distribution toward higher scores in the more food insecure group compared to the less food insecure group at follow‐up (Figure 3).

**FIGURE 4:**
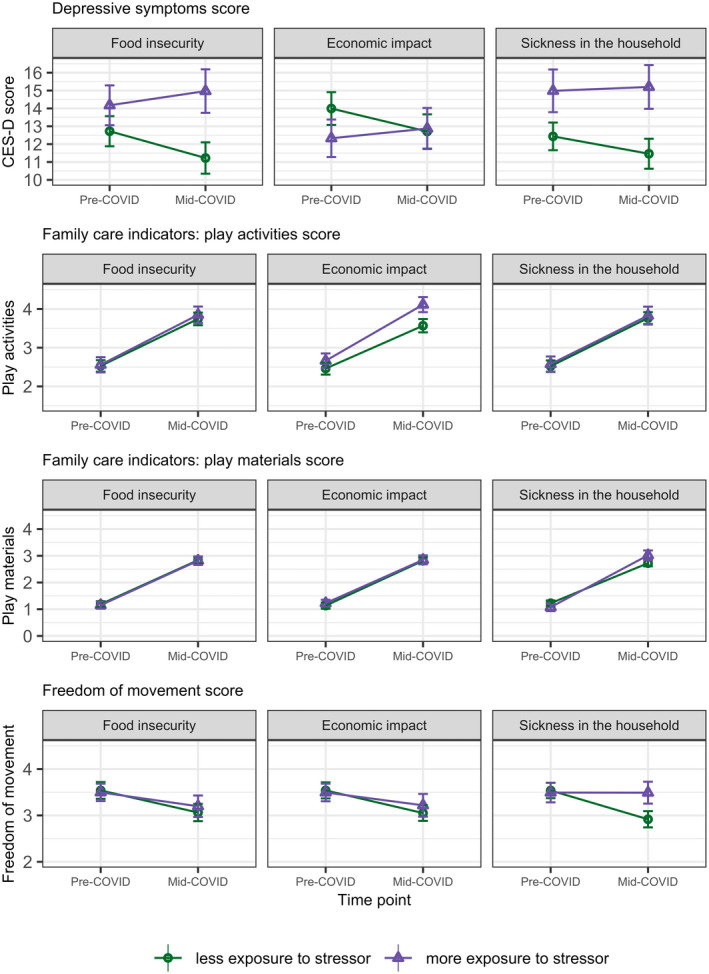
Difference‐in differences plots for CES‐D score, play activities, play materials, and freedom of movement outcomes over time, stratified by experiences at mid‐COVID timepoint. Figure presents matrix of results for twelve difference‐in‐differences analyses whereby three different exposures during the mid‐COVID time point (columns: food insecurity; economic impact; sickness in household) are related to four outcomes (rows: CES‐D; play activities; play materials; freedom of movement) during the mid‐COVID time period. "More exposed to food insecurity" = had two or more indicators of food insecurity. "More exposed to economic impact" = had both the primary earning member of the household lose their job and reduced household income since April 2020. "More exposed to sickness in the household" = had one or more household member experience COVID‐like symptoms since April 2020. CES‐D, Center for Epidemiologic Studies Depression scale

Depressive symptoms increased more between the pre‐ and mid‐COVID timepoints for those experiencing more food insecurity or a loss of income and job for the primary earner of the household during the mid‐COVID assessment, after adjustment for covariates at the child, caregiver, and household levels (average increases in CES‐D scores of 2.29, 95% CI 0.72 to 3.86 and 1.93, 0.31 to 3.35, respectively; Table 5; Figure 4).

**TABLE 5:** Results from difference‐in‐differences analysis in the cohort group who are more and less affected on the food security, economic, and health domains at follow‐up

Outcome	Exposure variable at the mid‐COVID assessment					
	More food insecure		Lost job and reduce income		Respondent or household members sick	
	DID estimate	*p*‐value	DID estimate	*p*‐value	DID estimate	*p*‐value
CES‐D Score	2.29 (0.72 to 3.86)	.004	1.83 (0.31 to 3.35)	.02	1.19 (−0.47 to 2.84)	.16
FCI Play activities	0.07 (−0.27 to 0.43)	.66	0.34 (0.01 to 0.68)	.05	0.01 (−0.35 to 0.37)	.96
FCI Play materials	0.02 (−0.26 to 0.29)	.91	−0.06 (−0.34 to 0.22)	.68	0.44 (0.16 to 0.73)	.002
Freedom of movement	0.17 (−0.17 to 0.53)	.32	0.21 (−0.14 to 0.57)	.24	0.62 (0.26 to 0.97)	.001

Note

DID estimate: Difference‐in‐differences estimates from a generalized estimating equation model accounting for repeated measures within participants, adjusted for village, child age category, maternal education, income, antenatal care, control over assets, household size, child sex, housing materials; FCI: Family Care Indicators, the play activities subscale is a sum score of the number of play activities that the caregiver participated in with the child in the previous 3 days (0–6); the play materials subscale is the number of different types of play materials the child has played with in the past 30 days (0–6); CES‐D: Center for Epidemiologic Studies 20 question Depression questionnaire, scores range from 0 to 60, with higher scores indicating more depressive symptoms experienced; Freedom of movement score is a sum score with one point for attending each of the following four places in the last 6 months, and an additional point if that location was attended alone: the market, medical facility, outside the village, paternal home or the home of a friend or a relative (0–8).

Caregiver depressive symptoms also increased more between timepoints when a household member was sick with COVID‐19‐like symptoms between April and July–September 2010 (average increase 1.19, 95% CI −0.47 to 2.84), although this difference was not significant at the *p* < .05 significance threshold. The greater the number of domains in which a caregiver was affected related to larger differences in CES‐D scores over time (Table S3).

For families who experienced a reduction of income and a loss of job for the primary earner in the household, there was an increase in reported play activities in the home between the pre‐ and mid‐COVID timepoints compared to families who did not experience this financial effect (0.34, 95% CI 0.01 to 0.68; Table 5; Figure 4). Having a household member sick was associated with a larger variety of play materials (0.44, 0.16 to 0.73) and more freedom of movement (0.41, 0.12 to 0.71) at follow‐up (Table 5; Figure 4). There were no other differences in outcomes between groups.

We found that the prevalence of CES‐D scores over 16 was higher among caregivers who experienced more impacts on food security, economic status, and health in the household at follow‐up (prevalence differences from difference‐in‐differences estimates: 0.06, −0.05 to 0.17; 0.06, −0.05 to 0.16; and 0.08, −0.03 to 0.19, respectively; results not shown). For the sensitivity analyses with caregiver depressive symptoms as an outcome re‐run with median regression all estimates were within 0.50 from the GEE estimates, and inference did not change for any analyses (Table S4). Inference also did not change for sensitivity analyses with caregiver freedom of movement as an outcome that were re‐run with a freedom of movement score that did not include questions about visiting a health facility (Table S5). A comparison of individuals in the full cross‐sectional sample at the mid‐COVID timepoint found similar results across most contrasts (Table S6).

## DISCUSSION

In this article, we report the widespread experiences of food insecurity and loss of employment and income among low‐income families in rural Bangladesh during the COVID‐19 pandemic, and the associated increased depressive symptoms among caregivers of young children. Specifically, primary caregivers had more depressive symptoms if they lived in families that experienced more food insecurity, both job loss of the main earner of the household and a loss of household income, or had sickness in the family after the countrywide lockdown due to the COVID‐19 pandemic, compared to those who did not have these experiences. Our findings relating to stimulating caregiving practices and freedom of movement were inconsistent, with more stimulating activities in the participants more affected by job loss and loss of income, more play materials and freedom of movement in the households that had at least one household member sick, and otherwise null results.

We found that 81% of the current sample reported any loss of household income and 10% reported a complete loss of income. These values are lower than those reported in a previous study in rural Bangladesh in which 96% of mothers had a reduction in paid work for the family and 39% experienced a complete loss of income (Hamadani et al., 2020). This difference may have been because the other study was conducted immediately after the lockdown when all income sources (factories, industries, offices, and transportation of agricultural products from rural to urban areas) were suddenly stopped. Other possible explanations for these differences are that the population in the earlier sample was more universally affected by the pandemic due to their proximity to the capital city Dhaka, and the effects on employment and income diminished over time, with our results from experiences in July–September, compared to earlier work done in May–June. Our results on changes in food habits to cope with financial losses are consistent with recent work from Bangladesh which found that in a cross‐sectional sample (*n* = 106) in Matlab, Bangladesh, in April and May 2020, 44% of the rural population sampled had consumed less food or changed their food habit to cope with financial losses due to the pandemic, compared to 48% in the current sample (Das et al., 2020).

We found that greater food insecurity mid‐COVID was present in families where the caregivers who had less than a primary school education, lived in a home without a permanent wall or concrete floor, were in the lowest income tertile, or had fewer assets. It may be that families who were better‐off financially were better able to use their resources to adapt to the sudden economic shutdown. Additionally, ownership of agricultural land and education of household members have been found to be associated with reduced food insecurity in Bangladesh (Raihan et al., 2018) and may have played a role in mitigating experiences of food insecurity and economic status for families during the COVID pandemic. Further, the availability and use of social and community support systems may have contributed to both financial and social support for caregivers and partially account for the heterogeneity in experiences of food security and financial impacts as well as mental health. Individual social capital has been linked to improved mental health outcomes, and in Indonesia, individual's trust in their community was found to be positively related to mental health independent of poverty (Ehsan & Silva, 2015; Tampubolon & Hanandita, 2014).

Between the pre‐COVID and mid‐COVID time points, depressive symptoms increased more in the groups affected on the food security, economic, and health domains at the mid‐COVID time point. The increases in depressive symptoms were largest when comparing caregivers who experienced more food insecurity with those who experienced less food insecurity. In a previous study set in Bangladesh, maternal mental health was assessed prior to the pandemic (between 2017 and February 2020) and in May–June 2020, and depressive symptoms scores were worse overall during the latter time point (Hamadani et al., 2020). Depressive symptoms in the other study were assessed with a modified seven‐question CES‐D scored using total number of days per week of symptoms reported as opposed to a Likert scale, and as such, their estimates cannot be compared directly to ours (Hamadani et al., 2020). A meta‐analysis including studies from high‐income countries published before July 5, 2020 found 3 studies that compared maternal mental health before and during the pandemic for post‐partum women, and found a pooled effect size of 0.40 (−0.05 to 0.96) for depressive symptom scores (Hessami et al., 2020). None of these studies were from LMICs, with populations from Italy, China, and Canada. In our analyses, we do not find increases in depressive symptoms overall, only within subgroups of caregivers who experienced more food insecurity and financial loss during the pandemic. When our estimate for the more food insecure group is converted to a measure of standard deviations of the population pre‐COVID, we find that the difference‐in‐differences estimate is 0.26 *SD* (0.08 to 0.44).

Previous work has linked poverty and food insecurity to poorer mental health (Lund et al., 2010; Tampubolon & Hanandita, 2014). Our analysis adds to this literature as well as the literature on the magnitude and duration of the effects of the pandemic and subsequent lockdowns on caregiver mental health, and suggests that examining effects in subgroups may be critical to understanding the burden of poor mental health. Participants in the less affected groups, on average, experienced improvement in depressive symptom scores over time. These improvements may have been partially a result of children getting older over time, and risk of depression in the postpartum time period being higher than risk of depression when children are older (Goodman, 2006).

We found that caregivers who had the primary earning member of their family lose a job and had reduced income due to the pandemic participated in a larger variety of play activities with their child mid‐COVID when comparing to those who had not. We speculate that job loss may have resulted in having more family members at home to help with household chores and thus may have allowed the primary caregiver to spend more time interacting with their young child. This may have reversed the expected relationship between increased maternal depressive symptoms and decreased stimulation in the home (Herba et al., 2016). We do not find any corresponding negative associations between compromised food security, economic, or health status and the variety of play activities that the caregiver participated in, or the variety of play materials in the home. The lack of correspondence between the results on maternal mental health and those on play activities may be both a result of both the increased number of family members at home during the pandemic and that our assessments of stimulation in the home measured the variety, but not the quantity or quality of stimulation. Though we did not find decreases in the variety of stimulating play activities participated in by the primary caregiver, and this may mitigate the overall effect of the pandemic on children, these interactions may have been more difficult for the caregiver to engage with due to their increased depressive symptoms, and may have been reduced in quality.

In families where one or more household member was sick with COVID‐like symptoms during the mid‐COVID assessment, there was a larger variety of play materials at home, and caregivers in these households had increased freedom of movement compared to households where no members were sick during this period. The latter relationship held even when travel to healthcare facilities was excluded from the freedom of movement score. Thus, it may be that the caregiver was required to leave the house for reasons related to the sickness or for tasks that would routinely fall on the sick household members, or that the symptoms were related to the caregiver having to participate in activities outside of the home. In the context of the COVID pandemic, the freedom of movement measure may not be a robust indicator of female autonomy.

The timing and duration of caregiver depressive symptoms matter for both caregiver and child well‐being, and the increase in depressive symptoms may have implications both for the child who was part of this study, as well as future children of the caregiver (Herba et al., 2016; Kurstjens & Wolke, 2001). Further, simultaneous effects on both economic status and caregiver mental health as are seen in this sample may be especially detrimental, as they perpetuate the cycle of poverty and poor mental health (Lund et al., 2011). Financial assistance or other means of economic empowerment could help ensure that families are able to meet their basic needs when their regular income has been compromised by the COVID‐19 pandemic. This support may be especially beneficial when targeted to families experiencing the greatest vulnerability, living remotely without access to other services. However, cash transfers and financial support face the same challenges faced by healthcare delivery, in that the most marginalized and poor populations are the hardest to reach, even when interventions are targeted (Golan et al., 2017; Mishra & Kar, 2017). In our sample, only 3% of the sample relied on government or NGO assistance to cover basic household needs, and this was higher (4%) among those who reported no income or some income but less than previously. Taken together, these findings suggest that either there is very little assistance offered in Chatmohar, or it is difficult to obtain for rural families.

As depicted in Figure 1, the *nurturing care framework* highlights that caregivers require an enabling environment including time, resources, and capabilities to provide nurturing care, and this environment is affected by policies, supporting services, and social contexts (Black et al., 2017; World Health Organization et al., 2018). The pandemic has affected the environment in which caregivers provide nurturing care, and the time, resources, and capabilities of caregivers have been affected. This research demonstrates how a lack of access to resources (financial and nutritional) has led to diminished capabilities (through worsened mental health), in line with the framework. The unavailability of financial support to families may result in long‐term effects on caregivers, children, and future generations.

We estimated the magnitude of effects that the COVID‐19 pandemic and subsequent lockdowns may have on population‐level child development outcomes through increased depressive symptoms among caregivers experiencing more food insecurity during the COVID pandemic. A meta‐analysis on the relationship between maternal depressive symptoms and child cognitive development found that children under 56 months whose mother's had postnatal depressive symptoms had −0.27 *SD*s (95% CI −0.43 to −0.11) lower cognitive scores (Liu et al., 2017). There are approximately 8.8 million children under 5 living in rural areas in Bangladesh (United Nations, Department of Economic, and Social Affairs, Population Division, 2018, 2019). If the proportion of children affected by increased food insecurity in the rural population of children under 5 in Bangladesh is the same as in our cohort sample (41.4%), an increase of caregiver depression of 6 percentage points in this group would represent over 218,000 children who are at increased risk of poor development. The effect size of −0.27 *SD*s on these 218,000 children would come close to reversing the effects of stimulation interventions to improve child development outcomes (*d* = .42, 0.36 to 0.48; Aboud & Yousafzai, 2015; Liu et al., 2017). This has the potential to set children back to a more disadvantaged starting point for future interventions, and have long‐term implications for their well‐being. As such, financial assistance or other means of economic empowerment could be pivotal to reducing the population impacts on child development and caregiver mental health.

A major strength of this study is the longitudinal sample, with baseline data collected in May–June, 2019, before the COVID‐19 pandemic. Another strength is that the information about household‐level experiences during the pandemic is available across multiple domains, and this information is used to stratify the sample and examine differences between subgroups with a difference‐in‐differences design. This approach is likely to be unbiased if the trends in outcomes over time due to unobserved factors are the same in each set of comparison groups.

Despite its strengths, the current study does have limitations. The greatest threat to validity is potential bias from loss‐to‐follow‐up in the cohort sample. We are optimistic about the validity of the sample, however, because the retained and lost samples are similar across all observed characteristics at the pre‐COVID assessment. In addition, we were not able to test the “parallel trends” assumptions for the differences‐in‐differences analysis, as we did not have data from additional prior time points to examine trends in outcomes prior to the pre‐COVID assessment. We address this concern by controlling for covariates that are theoretically related to the outcomes and differed during the pre‐COVID time point across groups. Further, as our follow‐up survey was done over the phone, we were not able to conduct any observations of the quality of caregiver‐child interactions or other observations of the home environment. A measure of the quantity and quality of stimulation in the home that included observations or other measures of the quality of home stimulation may have been more sensitive to changes in the caregiving environment. Finally, we did not conduct qualitative work on participants’ experiences during the COVID pandemic to complement and further examine the nuances in our quantitative results.

Future work should address the critically important range of other outcomes in addition to depressive symptoms that comprise mental health. Anxiety was assessed cross‐sectionally during May and June 2020 in Bangladesh, and it was found that 14% of participants reported moderate or severe anxiety, and when asked about if their symptoms had changed since the lockdown began, 99% stated that they experienced increased anxiety (Hamadani et al., 2020). The meta‐analysis by Hessami et al. found three studies that looked at anxiety scores pre‐ versus during the pandemic and found an increased in anxiety scores post with a mean difference of 0.82 (0.49 to 1.16; Hessami et al., 2020). Therefore, there are some mental health effects that we did not capture, and the full impact on caregiver mental health is likely to be larger than what was captured in this study. Additionally, future work on the concordance between the variety, quantity, and quality of child stimulation for caregivers with increased depressive symptoms would be helpful to further investigate our results for reported variety of stimulating activities and materials. Further, future work employing a mixed‐methods approach including qualitative data collection to explore attitudes, behaviors, and norms will contribute to a better understanding of experiences during the pandemic. Also, follow‐up work to understand the duration of the adverse conditions experienced during the COVID pandemic will contribute to the understanding of the magnitude and duration of impact on children's development. Finally, in addition to financial assistance, interventions that focus on caregiver mental health and the quality of responsive and nurturing care during times of crisis may be helpful in supporting children and caregivers and mitigate the negative impacts of the COVID‐19 pandemic in rural Bangladesh.

## CONCLUSION

We add to the existing literature the effects of the COVID‐19 pandemic and subsequent lockdowns on children 6–27 months through a more nuanced understanding of the effects of the pandemic on caregiver depressive symptoms, and estimate the potential impact this may have on child development outcomes. We do not find a consistent association between food security, loss of job and income, or sickness in the household during COVID‐19 directly on the variety of play materials or play activities in the home.

The enabling environment for nurturing care has been affected by the COVID‐19 pandemic, through increased depressive symptoms in families experiencing more financial loss and indicators of food insecurity. These changes did not correspond with decreased variety of play activities or play materials with young children in our study. Nonetheless, changes in caregiver mental health and household resources are likely to contribute to altered child development through pathways other than the variety of stimulating activities and materials including caregiver sensitivity and responsiveness, responsive feeding, and child health and nutrition. Based on previous work, we have estimated the magnitude of children that may have had their developmental trajectories altered through increased depressive symptoms in their caregivers, highlighting the magnitude of potential impact through these pathways.

Future work on the concordance between the variety, quantity, and quality of child stimulation for caregivers with increased depressive symptoms would be helpful to further investigate our results for reported variety of stimulating activities and materials. Our research underscores the urgency of financial and mental health interventions in rural Bangladesh to mitigate the long‐term effects of the COVID‐19 pandemic on caregivers and children, promote positive developmental trajectories, and improve later life outcomes.

## ETHICAL APPROVAL

Ethical approval was obtained from icddr,b, and the University of California, Berkeley.

## Supporting information

Supplementary Material
