# Supplementary material for: Effects of the COVID‐19 pandemic on caregiver mental health and the child caregiving environment in a low‐resource, rural context
Source: Child Dev. 2021 Sep 7;92(5):e764–80. doi: 10.1111/cdev.13651 (PMC8653040; doi:10.1111/cdev.13651)
Supplement: Supplementary file 1 — Supplementary Material [file CDEV-92-e764-s001.pdf]

SUPPLEMENTARY MATERIAL

Title: Effects of the COVID-19 pandemic on caregiver mental health and the child caregiving environment in a low-resource, rural context

Description of multistage sampling

The stages of sampling were as follows: 1) 109 villages were allocated across the 11 rural unions of Chatmohar according to the proportion of the population that lived in each union according to the most recent national census, with 5-13 villages allocated to each union; 2) Using data from a household listing carried out by our study team, all villages with under 25 households with children 6-24 months of age were merged with the adjacent village so all villages had at least 25 eligible individuals; 3) Population proportional to size sampling was done within each union to select the specific villages to sample from; 4) In each sampled village 15 primary caregivers were selected using stratified random sampling with replacement (if participants refused or were unavailable) in the following three age ranges: 6-12 months (n=7), 12-18 months (n=4) and 18 to 24 months (n=4).

Supplementary Figure 1a. Study sample including cross-sectional mid-COVID sample

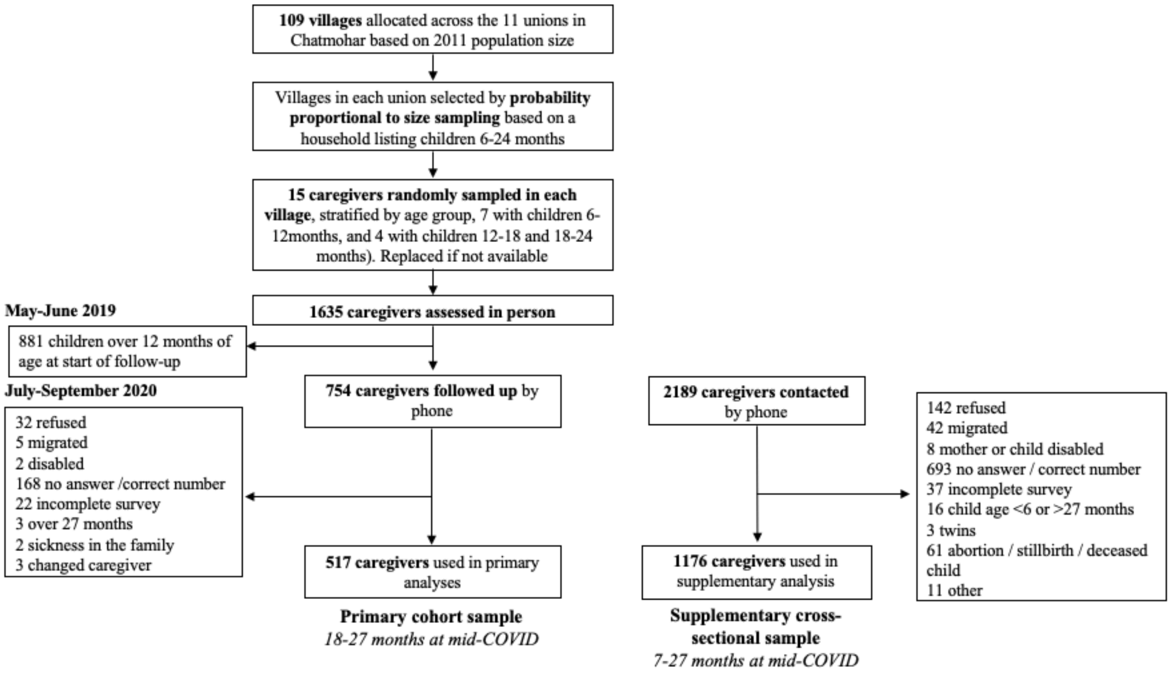

Note: In the cross-sectional sample participants from additional villages were recruited for the cross-sectional assessment when all of the eligible participants in the original villages were attempted to be surveyed, and the desired sample size had not yet been met.

Supplementary Figure 1b. Graphical depiction of samples used in primary and supplementary analyses

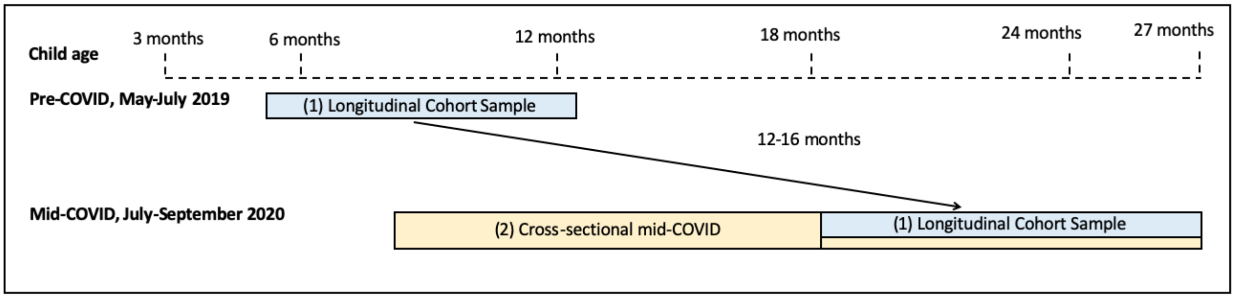

Development of COVID-19 related questions

The research team first conducted qualitative interviews with 7 mothers of children 24-36 months of age in Chatmohar to better understand the ways in which the COVID-19 pandemic and subsequent mitigation strategies had affected their lives. We used parts of a survey previously done in Bangladesh to gather information on COVID-19 and added on some additional questions to address the specific concerns from the qualitative work. We then piloted this adapted survey with 15 caregivers of children 6-24 months to test for clarity and length

Supplementary Table 1. COVID-19 related questions

	**RINEW-G Module 23: Impact of COVID-19**	
****Section A: Interaction and movement of the target child****		
q2301	2301. Since the shutdown/lockdown due to COVID-19, have there been any changes in the number of people staying in your household (this number is the total number of household members who usually lived/stayed most of their time in that household)?	1. Yes<br>0. No
q2302	2302. How has it changed?<br><br>If the response is 4, then say to the mother, "I am sorry to hear that"	1. Increased; an adult or child has moved in<br>2. Increased; new baby member has been born<br>3. Decreased; an adult or child has left<br>4. Decreased; a household member has passed away
q2302a	2302a. How long an additional member (household members/relatives) is staying or stayed in your household? **Record the duration in days**	
q2302b	2302b. Is the additional member (household member/relative) still staying in your household?	1. Yes<br>0. No

q2303	2303. What was the cause of death? ***(Free response. Do not read options. Select all that apply)***	1. COVID-19<br>2. Fever<br>3. Cold<br>4. Difficulty in breathing<br>5. Accident/Injury<br>6. Stroke<br>77. Others (please specify)<br>99. Don't know
q2303_ot	q2303_ot. Others (please specify)	77. Others (please specify)
q2304	2304. Did the household member die at a health facility?	1. Yes<br>0. No
q2305	2305. For the last 7 days, how much have you interacted (talking, playing, or spending time) with your child compared to time before lockdown due to COVID-19?	1. Same<br>2. More<br>3. Less
q2306	2306. Why have you interacted (talking, playing, or spending time) with your child more/less? (Select all that apply)	1. Spending more time in the household due to restricted movement outside the house after lock down due to COVID-19<br>2. Child's sickness<br>3. Own sickness<br>4. More Household chores<br>5. Less household chores<br>77. Others (please specify)
q2307	2307. For the last 7 days, how much total time has your child interact with (talking, playing, or spending time) other household members compared to time before lockdown due to COVID-19?	1. Same<br>2. More<br>3. Less
q2308	2308. Why other household members interacted (talking, playing, or spending time) with your child more/less?	1. Spending more time in the household due to restricted movement outside the house after lock down due to COVID-19<br>2. Child's sickness<br>3. More family members in the household since the lockdown due to COVID-19<br>4. Due to mother’s sickness other family members spending more time with the child<br>77. Others (please specify)

q2309	2309. What changes have been made in the household due to the COVID-19 pandemic? (Do not read the responses. Select all that apply)	0. No change due to COVID-19<br>1. More handwashing<br>2. More cleaning of household and outdoor space<br>3. Interacting less with people outside the household<br>4. Restricted movement<br>5. Wearing a mask when going outside<br>6. Children have stopped going to school / Madrasa<br>77. Others (please specify)
****Section B: Food security and household food consumption****		
q2311	2311. Was your household able to buy essential food items over the past 7 days?	1. Yes<br>0. No
q2312	2312. Which essential food items were you unable to obtain?<br>***Read all the options, select all that apply***	1. Water<br>2. Rice<br>3. Lentils<br>4. Oil<br>5. Fresh Fish<br>6. Chicken<br>7. Beef<br>8. Egg<br>9. Vegetables<br>77. Other (please specify)
q2313	2313. Why were you unable to buy these items?<br>***Read out all options, select all that apply***	1. Items were not available<br>2. Items were more expensive than usual<br>3. Items were the same cost but household had less money to spend<br>4. Markets/shops were closed<br>0. None of the above
q2314	2314. In the last 7 days, are you consuming the same, more, or less food when compared to same time last year?	1. Same<br>2. Reduced<br>3. Increased
q2315	2315. Why reduced?	1. Due to COVID-19 pandemic<br>2. Due to own sickness<br>77. Others (please specify)
****Section C: Health status of the household****		
q2321	2321. Have you been sick since the lockdown due to COVID-19?	1. Yes<br>0. No

q2322	2322. What kind of sickness?<br>***Select all that apply***	1. Fever<br>2. Cough<br>3. Cold<br>4. Loss of taste/smell<br>5. Shortness of breath or had difficulty in breathing<br>6. COVID-19<br>77. Others (please specify)
q2323	2323. Did you go to a health facility for treatment?	1. Yes<br>0. No
q2324	2324. Why didn't you go to the health facility for treatment?<br>***Do not read out the options. Select all that apply***	1. No facility was open<br>2. Facility was not officially closed but (we had heard) there was no staff<br>3. We were afraid of catching COVID-19 (or other illness) at the health facility<br>4. Too expensive<br>5. Sickness was possible to manage at home<br>77. Others (please specify)
q2325	2325. Did any other members of your household fall sick since the lockdown due to COVID-19?	1. Yes<br>0. No
q2325a	2325a. Who fell sick?	1. Target child<br>2. Other child<br>3. Mother/Mother In Law<br>4. Father/Father In Law<br>5. Close Relative<br>77. Other (specify)
q2326	2326. What kind of sickness?<br>***Select all that apply***	1. Fever<br>2. Cough<br>3. Cold<br>4. Loss of taste/smell<br>5. Shortness of breath or had difficulty in breathing<br>6. COVID-19<br>77. Others (please specify)
q2327	2327. Did they go to a health facility for treatment?	1. Yes<br>0. No
q2328	2328. Why didn't they go to the health facility for treatment?<br>***Do not read out the options. Select all that apply***	1. No facility was open<br>2. Facility was not officially closed but (we had heard) there was no staff<br>3. We were afraid of catching COVID-19 (or other illness) at the health facility<br>4. Too expensive<br>5. Sickness was possible to

		manage at home<br>77. Others (please specify)
q2329	2329. Have there been any deaths in your household since the lockdown due to COVID-19 pandemic?<br><br>***If the response is “Yes” then please say the mother, “I am really sorry to hear that.”***	1. Yes<br>0. No
q2329a	2329a. Who was it?	1. Target child<br>2. Other child<br>3. Mother/Mother In Law<br>4. Father/Father In Law<br>5. Close Relative<br>77. Other (specify)
q2330	2330. What was the cause of death?<br>***Free response. Do not read options. Select all that apply***	1. COVID-19<br>2. Fever<br>3. Cold<br>4. Difficulty in breathing<br>5. Accident/Injury<br>6. Stroke<br>77. Others (please specify)<br>99. Don't know
q2331	2331. Did death occur at a health facility?	1. Yes<br>0. No
**Section D: Household economic status**		
q2341	2341. Did the employment type of any of your household members change since the shutdown/lockdown due to COVID-19 started?	1. Yes<br>0. No
q2342	2342. What type of changes happened?<br>***Do not read out the options. Select all that apply***	1. No job/income source of the main earning member of the household<br>2. Earning from a temporary source by the main earning member of the household<br>3. Main earning member of the household become a day laborer<br>4. Other members of the household started earning from a temporary source<br>5. Les s work/decreased salary for household members<br>77. Others (please specify)
q2343	2343. What is the status of your household income since April 2020?	1. No income<br>2. Some income, but less than previous income<br>3. Same as previously<br>4. Earning more than previous

q2344	2344. In the past 7 days, did you or your household members use any of the following to cover your household's basic needs?<br>***Readout all options, select all that apply***	1. Look for ways to earn additional money (e.g., work more hours, do an occasional job, etc.)<br>2. Reduce the number or size of meals for some household members<br>3. Rely on less preferred and less expensive foods<br>4. Use cash or bank savings<br>5. Sell assets<br>6. Borrow food or ask for help from a friend or relative or neighbor<br>7. Rely on Government or NGO assistance<br>8. Donations<br>9. Taken loan from someone else<br>77. Others (please specify)<br>0. None of the above
**Section E: Other impacts**		
q2351	2351. Now I would like to ask, if any other experience related to COVID-19 you want to share with me?	

Supplementary Table 2. Comparison of cohort sample assessed vs. lost to follow-up

	Sample, n (%) or mean ± SD	
	Assessed (n=520)	Lost (n=234)
**Caregiver Characteristics**		
Completed primary education (6+ years)	69% (369)	62% (144)
Currently pregnant	1% (6)	1% (3)
Number of children < 15 yrs under care	1.9 ± 0.8	1.9 ± 0.80
Muslim	98% (510)	97% (227)
CES-D score (0-60)	13.4 ± 8.7	14.2 ± 9.4
Mobility score (0-8)	3.5 ± 1.6	3.6 (1.73)
**Child Characteristics**		
Age (in months)	8.6 ± 1.8	8.5 ± 1.9
Female	51% (263)	52% (122)
FCI Play activities subscale (0-6)	2.5 ± 1.5	2.4 ± 1.6
FCI Play materials subscale (0-6)	1.2 ± 1.1	1.1 ± 1.0
1+ children's book(s) present in home	3.7% (19)	1.7% (4)
**Household Characteristics**		
Household size	5.2 ± 1.8	5.3 ± 1.9
Has cement floor	19% (98)	15% (35)
Has brick walls	23% (123)	20% (49)
Has refrigerator	22% (112)	18% (41)

Supplementary Table 3. Difference-in-differences analyses stratified by sum of impact across food security, economic and health domains (cohort sample)

sum of impact across food security, economic and health	n	DID estimate	
**0**	147	Ref	
**1**	187	0.38 (-1.40 to 2.17)	p=0.673
**2**	135	1.56 (-0.51 to 3.60)	p=0.14
**3**	51	5.41 (2.58 to 8.24)	p=<0.001
**2+**	186	2.61 (0.71 to 4.50)	p=0.007

Supplementary Table 4. Sensitivity analyses: Median regression for CES-D score outcome

Exposure variable:	More food insecure	Lost job and reduce income	Respondent or household members sick
**Median CES-D**	1.85 (0.62 to 3.08)	1.33 (0.05 to 2.60)	1.98 (1.13 to 2.83)

Supplementary Table 5. Sensitivity analyses: Difference-in-differences estimates with caregiver freedom of movement score not including health clinic

Exposure variable:	More food insecure	Lost Job and Reduce Income	Respondent or household members sick
**Freedom of movement not including health clinic (0-6)**	0.15 (-0.15 to 0.44) p=0.30	0.14 (-0.16 to 0.44) p=0.36	0.41 (0.12 to 0.71) p=0.006

Difference-in-differences estimates from a generalized estimating equation model accounting for repeated measures within participants, adjusted for village, child age category, maternal education, income, antenatal care, control over assets, household size, child sex, housing materials; FCI: Family Care Indicators, the play activities subscale is a sum score of the number of play activities that the caregiver participated in with the child in the previous three days (0-6); the play materials subscale is the number of different types of play materials the child has played with in the past week (0-6) CES-D: Center for Epidemiologic Studies 20 Question Depression questionnaire, scores range from 0-60, with higher scores indicating more depressive symptoms experienced; Freedom of movement score is a sum score with one point for attending each of the following four places in the last 6 months, and an additional point if

that location was attended alone: the market, medical facility, outside the village, paternal home or the home of a friend or a relative (0-8)

Supplementary Table 6. Cross-sectional differences in child-development risk factors by exposure status in the full mid-COVID sample

Exposure variable:	More food insecure	Lost job or reduce income	Respondent or household members sick
**CESD-Score**	3.48 (2.66 to 4.30)	2.03 (1.24 to 2.82)	2.75 (1.81 to 3.68)
**Play activities**	0.21 (0.05 to 0.37)	0.38 (0.24 to 0.51)	0.19 (0.04 to 0.33)
**Play materials**	0.11 (-0.00 to 0.22)	-0.04 (-0.14 to 0.06)	0.23 (0.09 to 0.37)
**Freedom of movement**	0.06 (-0.08 to 0.21)	0.17 (-0.02 to 0.36)	0.58 (0.41 to 0.74)

Estimates are mean differences (95% CIs) from a generalized linear model with standard errors clustered by village. The comparison group for each column is those who did not experience the stated exposure at the mid-COVID time point. Differences are adjusted for child age (2 month categories), maternal education (category), income (tertile), antenatal care (4+ vs <4), control over assets (binary), household size, child sex, housing materials (floor and roof). Estimates represent mean differences between respondents more and less affected on each domain during the mid-COVID assessment.
n=1,685 due to missing data on covariates for 11 individuals
